# Leaves and Spiny Burs of *Castanea Sativa* from an Experimental Chestnut Grove: Metabolomic Analysis and Anti-Neuroinflammatory Activity

**DOI:** 10.3390/metabo10100408

**Published:** 2020-10-13

**Authors:** Ilaria Chiocchio, Cecilia Prata, Manuela Mandrone, Fortuna Ricciardiello, Pasquale Marrazzo, Paola Tomasi, Cristina Angeloni, Diana Fiorentini, Marco Malaguti, Ferruccio Poli, Silvana Hrelia

**Affiliations:** 1 Department of Pharmacy and Biotechnology, Alma Mater Studiorum—University of Bologna, Via Irnerio 48, 40126 Bologna, Italy; ilaria.chiocchio2@unibo.it (I.C.); cecilia.prata@unibo.it (C.P.); paola.tomasi3@unibo.it (P.T.); diana.fiorentini@unibo.it (D.F.); ferruccio.poli@unibo.it (F.P.); 2 Department for Life Quality Studies, Alma Mater Studiorum—University of Bologna, Corso d’Augusto 237, 47921 Rimini, Italy; fortun.ricciardiello@studio.unibo.it (F.R.); pasquale.marrazzo2@unibo.it (P.M.); marco.malaguti@unibo.it (M.M.); silvana.hrelia@unibo.it (S.H.); 3 School of Pharmacy, University of Camerino, Via Gentile III da Varano, 62032 Camerino, Italy; cristina.angeloni@unicam.it

**Keywords:** ^1^H NMR-based Metabolomics, neuroinflammation, flavonoids, *Castanea sativa*, waste valorization

## Abstract

*Castanea sativa* cultivation has been present in Mediterranean regions since ancient times. In order to promote a circular economy, it is of great importance to valorize chestnut groves’ by-products. In this study, leaves and spiny burs from twenty-four *Castanea* trees were analyzed by ^1^H NMR metabolomics to provide an overview of their phytochemical profile. The Orthogonal Projections to Latent Structures Discriminant Analysis (OPLS-DA) performed on these data allowed us to distinguish ‘Marrone’ from ‘Castagna’, since the latter were generally more enriched with secondary metabolites, in particular, flavonoids (astragalin, isorhamnetin glucoside, and myricitrin) were dominant. Knowing that microglia are involved in mediating the oxidative and inflammatory response of the central nervous system, the potential anti-inflammatory effects of extracts derived from leaves and spiny burs were evaluated in a neuroinflammatory cell model: BV-2 microglia cells. The tested extracts showed cytoprotective activity (at 0.1 and 0.5 mg/mL) after inflammation induction by 5 µg/mL lipopolysaccharide (LPS). In addition, the transcriptional levels of IL-1β, TNF-α, and NF-kB expression induced by LPS were significantly decreased by cell incubation with spiny burs and leaves extracts. Taken together, the obtained results are promising and represent an important step to encourage recycling and valorization of chestnut byproducts, usually considered “waste”.

## 1. Introduction

*Castanea sativa* Mill. (*Fagaceae*) is widespread in the Mediterranean region, where, since ancient times, its cultivation for timber and nut production has played a pivotal role in local sustenance and economy. In the last decade, increasing attention has been given to the chestnut waste that is generated yearly, which has a negative impact on both the environment and economy [1,2]. In fact, farmers tend to burn spiny burs, a maintenance practice, which could be avoided by adopting more sustainable solutions [1,2,3]. In this context, the implantation of circular economy practices, based on valorization of a crop’s waste material, plays a pivotal role. Regarding chestnut by-products, Costa-Trigo and co-workers suggested the application of chestnut burs extract for the production of culture media suitable for the growth of a wide range of microorganisms [4]. Moreover, chestnut shells of an Italian cultivar “Marrone di Roccadaspide” PGI (Protected Geographical Indication) were found to be endowed with antioxidant activity linked to their high content of tannins [5]. The abundance of total phenols and hydrolysable tannins confers to chestnut grove by-products interesting anti-inflammatory activity, as demonstrated by the ability of *Castanea* shell extracts to reduce the levels of cytokines and other biomarkers of inflammation in several experimental models [6,7,8,9,10,11].

The present study was carried out on leaves and spiny burs of *Castanea sativa* collected from trees growing in the experimental chestnut grove of Granaglione, situated on the Apennines Mountains in Emilia-Romagna [12,13], where a number of different cultivars of *C. sativa*, are cultivated and studied.

In order to first obtain an overview of the phytochemical composition of the samples, their ^1^H NMR metabolomic profiles were measured.

Metabolomics relies on untargeted analysis protocols handled with multivariate data treatment. This workflow has already been applied successfully in several areas of research, from human diagnostics and epidemiology to the plant sciences [14]. In this latter field of study, metabolomics was successful at facilitating the identification of the active components of medicinal plants [15], studying plant ecotypes and biological features [16,17], and controlling food and botanical quality, both in terms of nutraceutical/biological properties and fraud detection [18,19].

Since the analyzed samples were classified as ‘Castagna’ and ‘Marrone’ by pomological analysis, the obtained metabolomic data were also treated by chemometrics in order to explore the occurrence of differences between these two groups.

Moreover, with the view of valorizing *Castanea* by-products, the potential cytoprotective and anti-inflammatory roles of leaves and spiny burs extracts were investigated. In fact, according to previous studies, *Castanea* by-products are able to decrease oxidative stress [20] and consequently they could be promising in counteracting chronic degenerative diseases. Inflammation represents a feature of all chronic degenerative diseases, among which neurodegenerative diseases are considered a real threat to human health. It has been suggested that a cascade of processes collectively called neuroinflammation, which involves support cells called glia, contributes to neurodegeneration [21]. In particular, the activation of the neuroimmune cells, microglia, into proinflammatory states is an effective endogenous defense that protects the central nervous system (CNS) against microorganisms and injuries. It is usually a positive mechanism that aims to eliminate threats and restore homeostasis [22]. However, chronically activated and proliferating microglia promote the neuroinflammatory state by releasing cytokines and reactive oxygen and nitrogen species, ultimately causing oxidative damage to the neurons [23]. Growing experimental evidence suggests that controlling microglia activation may have protective effects against neurodegenerative diseases [24].

Therefore, the most phytochemically diverse samples, according to metabolomic analysis, were tested in a cellular model of microglia (BV-2 cells) to evaluate their potential neuroprotective and anti-inflammatory activities.

Hence, the overall objective of this study was to upgrade phytochemical knowledge on *Castanea* and its biological properties, with a particular attention to provide a basis for the valorization of chestnut grove waste material.

## 2. Results and Discussion

### 2.1. Metabolomic Analysis

**Figure 1:**
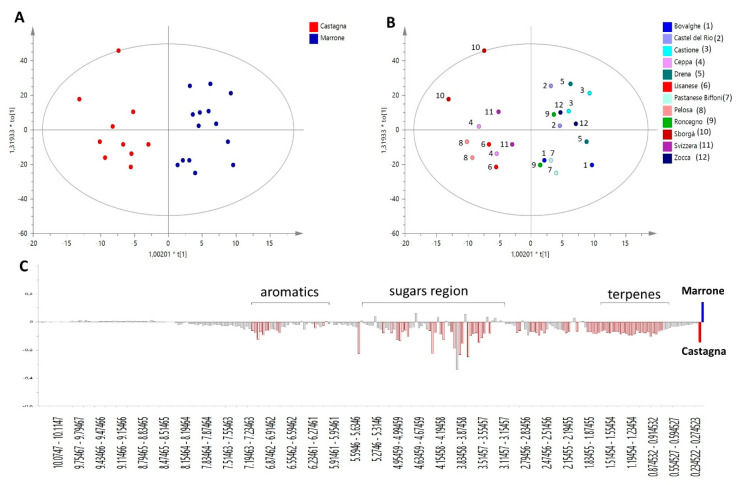
OPLS-DA model performed on ^1^H NMR profiles of different *C. sativa* cultivars, discriminating ‘Castagna’ and ‘Marrone’ on the basis of leave metabolome. (**A**) Score scatter plot colored according to model’s given classes; (**B**) Score Scatter plot colored according to the different cultivars analyzed (reported in the legend), each cultivar is represented by samples collected from two different trees; (**C**) Predictive loading plot is a schematic representation of bucketed ^1^H NMR spectra, indicating the most important ^1^H NMR signals contributing to the discrimination of ‘Castagna’ (in red on the negative axis of the plot) from ‘Marrone’ (on the positive axis of the plot).

In order to compare the phytochemical profiles of ‘Castagna’ and ‘Marrone’, the Orthogonal Projections to Latent Structures Discriminant Analysis (OPLS-DA) was built. This is a powerful multivariate data modeling tool that provides insights into separations between experimental groups based on high-dimensional spectral measurements, i.e., from NMR. In this case, it was built using bucketed ^1^H NMR spectra as the *x* variables, and ‘Castagna’ and ‘Marrone’ (Figure 1A) as the discriminant classes, as identified by pomological analysis. Three components maximized the explained 87.5% of the variation in the data set (given by *R*^2^*x*(Cum)), *R*^2^*y*(Cum) was 84.8%, while the obtained *Q*^2^(Cum) was 73.1%, indicating good model predictability (*Q*^2^ must be equal or higher than 50%). The model was further validated by the permutation test, giving *R*^2^(Cum) = 84.8% and *Q*^2^(Cum) = 73.1%, and CV-ANOVA resulting in *p* = 0.41 × 10^−3^ and *F* = 7.68. The overall parameters proved that the developed model was not only interpretable but also predictive and, thus, able to discriminate ‘Castagna’ from ‘Marrone’ on the basis of the leaves’ phytochemical profile.

It is worth noting that for each class, samples collected from two different trees of the same specific cultivar were analyzed, and they generally showed a reproducible metabolome, indicated by their closeness in the score of the scatter plot of the model (Figure 1B).

*S*-plot, loading plot, and VIP (Variable Influence on Projection) plot (not shown) of the OPLS-DA model explain the relationships between *x* variables (^1^H NMR signals) and given classes, in this case, ‘Castagna’ and ‘Marrone’. Thus, these plots were used to provide information on metabolites peculiar to a specific *Castanea* cultivar and important for its metabolomics-based distinction and identification.

‘Marrone’ leaves were characterized by small amounts of all metabolites, as highlighted by Figure 1C, where the highest intensity of the general spectral signals was found for ‘Castagna’ samples. Glucose and quinic acid were the only two metabolites showing a slight increasing trend in ‘Marrone’, but based on standard deviation calculated on VIP plot results, it is clear that this observation is not generalizable for all ‘Marrone’ samples; therefore, these metabolites are not trustworthy markers of distinction for ‘Marrone’.

Specifically, several aromatic signals were found to be less concentrated in ‘Marrone’, among them, two doublets at δ 5.95 and 6.03 with coupling constants around 2.2 Hz, which are generally characteristic of flavonoid protons situated on aromatic ring B [25]. They increased linearly with other aromatic signals and a doublet at δ 0.8, which is potentially related to the methyl group of rhamnose that is a common sugar moiety of several glycosylated flavonoids.

The results obtained from this model made essential further studies aimed at characterizing the main flavonoids contained in leaves.

**Figure 2:**
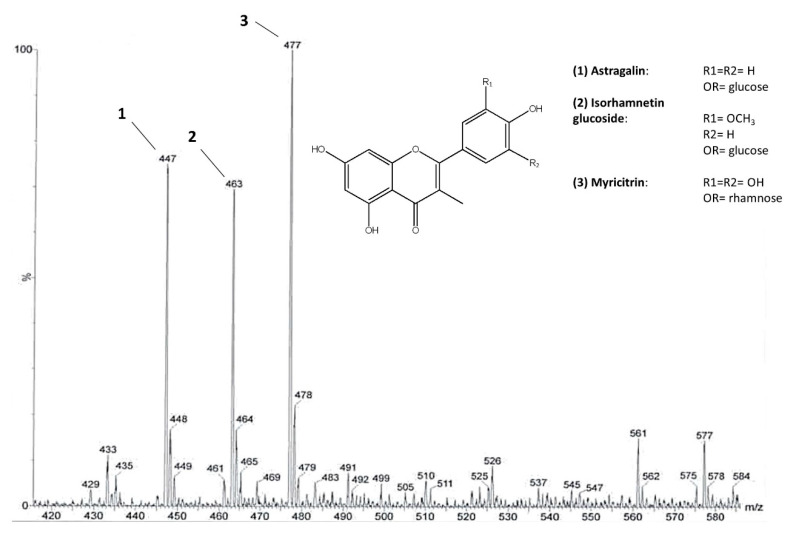
The main flavonoid glycosides identified in *C. sativa* leaves. ESI-MS spectrum of the flavonoid fraction obtained after direct infusion. Molecular ions at *m*/*z* 447, 477, and 463 belong to astragalin (1), isorhamnetin glucoside (2), and myricitrin (3), respectively.

The highest content of flavonoids was revealed, by ^1^H NMR analysis, in the EtOAc fraction that was derived from the liquid–liquid partition of an extract obtained by pooling all samples. Thus, this fraction was further fractionated by column chromatography. Three main flavonoids were chemically characterized through NMR and MS analysis. Mono and bi-dimensional NMR experiments allowed to elucidate the substitution pattern of the B ring (Table S1 and Figures S1–S4), while MS provided the molecular weights. On this basis, the identified flavonoids were astragalin [M-H]^−^ ion at *m*/*z* 447, isorhamnetin glucoside [M-H]^−^ ion at *m*/*z* 477, and myricitrin [M-H]^−^ ion at *m*/*z* 463 (Figure 2). The presence of astragalin and isorhamnetin in *C. sativa* leaves was already reported [26]. Moreover, astragalin was also found in *C. sativa* burs and flowers [27,28] and isorhamnetin glucoside was found in flowers [29]. Various glycosides of myricetin, kaempferol, and isorhamnetin were also found in *C. sativa* and *C. crenata* flowers [28,29,30].

**Figure 3:**
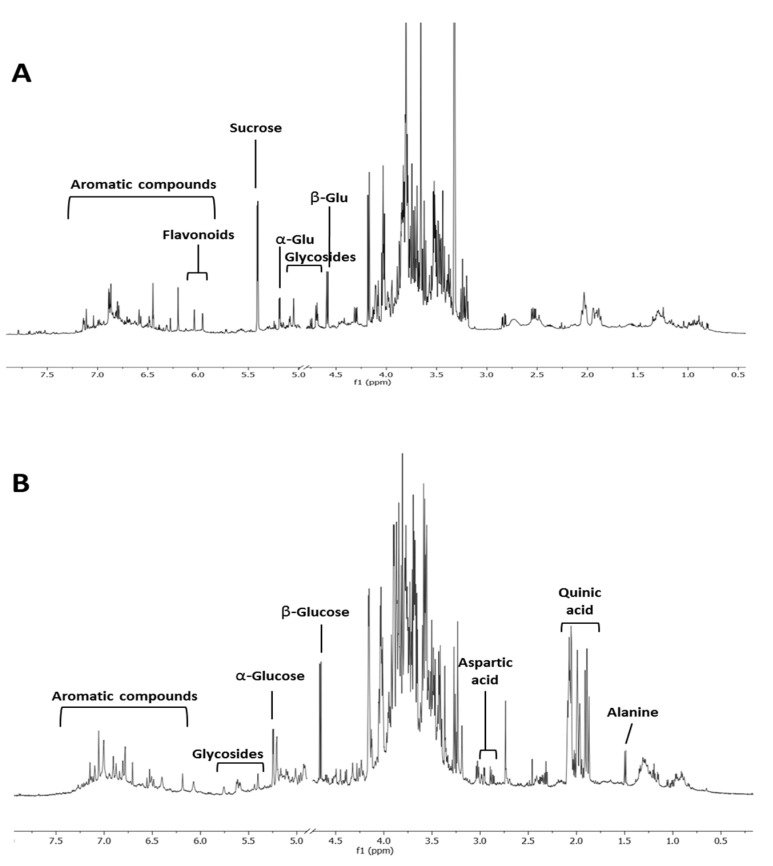
^1^H NMR profiling of *C. sativa* of representative leaves (**A**) and spiny burs (**B**) hydroalcoholic extract.

Spiny burs hydroalcoholic extracts were investigated through ^1^H NMR profiling. In contrast to the variation seen in the extracts from leaves, no specific metabolomics variation could be associated to ‘Castagna’ and ‘Marrone’ spiny burs. Compared to leaves (Figure 3A), this organ was less rich in secondary metabolites. Glucose and quinic acid were the most abundant compounds (Figure 3B). However, the aromatic region of the spectrum also showed numerous signals, potentially ascribable to tannins, which were previously reported to be contained in this plant material [27].

### 2.2. Effects of Chestnut Extracts on Cell Viability in Microglia BV-2 Cells

Since it has been demonstrated that the BV-2 microglia cell line is a valid model system to study inflammation, and its response to lipopolysaccharide (LPS) is comparable to that of primary microglia [31], this cell model was chosen to study the potential anti-inflammatory and neuroprotective role of different extracts from chestnut by-products.

**Figure 4:**
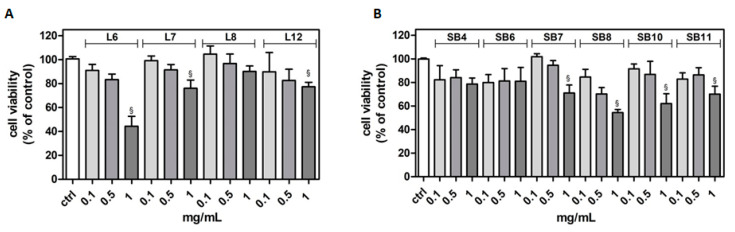
Effect of different extracts from chestnut by-products on the viability of BV-2 cells. BV-2 cells were incubated for 24 h with increasing concentrations (0.1, 0.5, 1.0 mg/mL) of extracts from chestnut leaves L (panel **A**) and spiny burs SB (panel **B**). Viability was evaluated by MTT test, as reported in the Materials and Methods section. Results are expressed as means ± SD of three independent experiments. Statistical analysis was performed by Bonferroni multiple comparison test following one-way ANOVA. ^§^ *p* < 0.05, significantly different from control cells.

The potential cytotoxicity of different extracts from leaves and spiny burs, obtained as described in the Materials and Methods section, was tested in the BV-2 cell line. Cells were incubated with increasing concentrations of extracts (0.1–1 mg/mL) for 24 h, and then their viability was evaluated by MTT assay (Figure 4). Results show that, in several cases, the highest concentration of extracts tested (1 mg/mL) significantly reduced BV-2 viability. Extract concentrations ranging from 0.1 to 0.5 mg/mL did not affect cell viability compared to control cells. Therefore, this range of concentration was used in the subsequent experiments in order to evaluate potential cytoprotective and anti-inflammatory activities exerted by the extracts.

### 2.3. Cytoprotective Effects of Chestnut Extracts in the Presence of Inflammatory Stress

**Figure 5:**
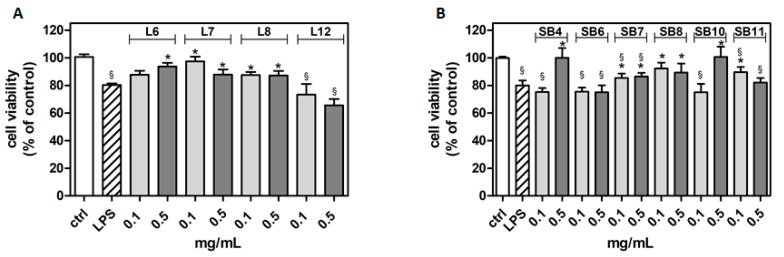
The effect of different extracts from chestnut by-products on the viability of BV-2 cells treated with lipopolysaccharide (LPS). BV-2 cells were incubated for 3 h with increasing concentrations (0.1, 0.5 mg/mL) of extracts from chestnut leaves L (panel **A**) and spiny burs SB (panel **B**), then 0.5 μg/mL LPS was added and the cells were incubated for a total of 24 h. Viability was evaluated by MTT test, as reported in the Materials and Methods section. Results are expressed as means ± SD of three independent experiments. Statistical analysis was performed by Bonferroni multiple comparison test following one-way ANOVA; ^§^ *p* < 0.05, significantly different from control cells; * *p* < 0.05, significantly different from LPS-treated cells.

To evaluate the possible cytoprotective role of the chestnut extracts against inflammatory stress, BV-2 cells were incubated with the different chestnut extracts in the presence or absence of LPS, as an inflammation inducer [32], and assessed for cell viability by MTT test, as reported in Figure 5.

The results show that many of the tested chestnut leaves extracts are able to exert a significant protective effect following LPS-generated inflammatory stress, with the exception of L12 (“Zocca”), which showed no significant differences from that of LPS-treated cells. Notably, according to the metabolomic analysis, L12 also showed a lower flavonoid content compared to that of the other samples tested. These data might suggest the importance of flavonoids in cytoprotective and anti-inflammatory activities, as it has been reported by Spagnuolo et al. [33]. However, other plant constituents, such as tannins and coumarins, might also counteract inflammation [34,35]. For instance, a coumarin endowed with anti-inflammatory activity was found in the inner shell of chestnuts (*Castanea crenata*) [36].

### 2.4. Anti-Inflammatory Effects of Chestnut Extracts

**Figure 6:**
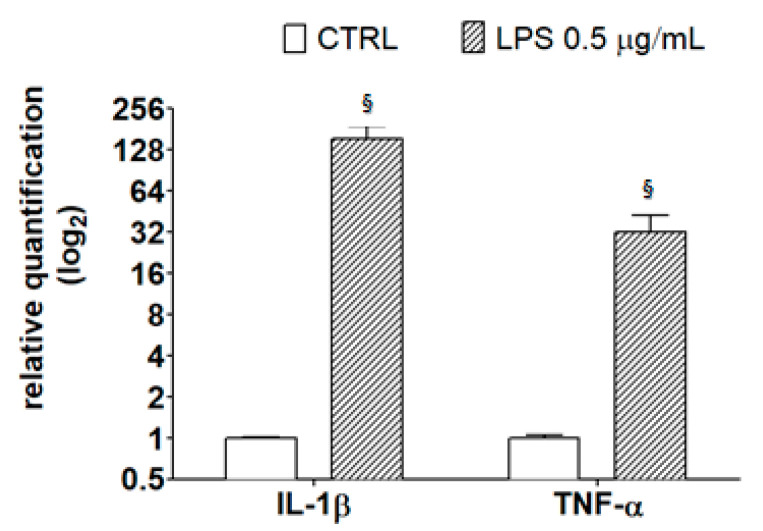
Transcriptional level of inflammatory markers in LPS-treated BV-2 cells. BV-2 cells were incubated with LPS (0.5 μg/mL) for 21 h; then, RNA was extracted, reverse-transcribed to cDNA, and analyzed by RT-PCR using specific primers for IL-1β and TNF-α, as described in the Materials and Methods section. Results are expressed as means ± SD of three independent experiments. Statistical analysis was performed by Bonferroni multiple comparison test following one-way ANOVA. ^§^ *p* < 0.05, significantly different from control cells.

As reported by Henn et al. [31], after exposure to LPS, BV2 cells show a broad response of gene activation, and many of the activated genes correspond to inflammatory mediators, such as IL-1β and TNF-α. These inflammatory mediators play an important role in the pathological processes of neurodegenerative diseases, as detailed in the review by Smith et al. [37]. To verify this observation in our model system, cells were treated with 0.5 μg/mL LPS for 21 h then subjected to RT-PCR analysis using specific primers for the detection of inflammatory markers. Results in Figure 6 confirm that LPS caused an increase in the transcriptional levels of IL-1β and TNF-α, highlighting the amplitude of the response of LPS-treated BV2 cells with respect to controls.

**Figure 7:**
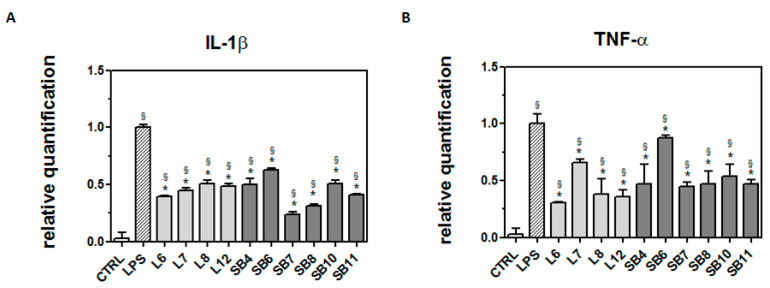
Protective effect of extracts from chestnut by-products on the transcriptional level of inflammatory markers in LPS-treated BV-2 cells. BV-2 cells were incubated for 3 h with 0.5 mg/mL extracts from chestnut leaves (L) and spiny burs (SB); then, 0.5 μg/mL LPS was added and the cells were incubated for a total of 24 h. Cells were then subjected to RNA extraction and analyzed by RT-PCR using specific primers for IL-1β (panel **A**) and TNF-α (panel **B**), as described in the Materials and Methods section. Results are expressed as means ± SD of three independent experiments. Statistical analysis was performed by Bonferroni multiple comparison test following one-way ANOVA. ^§^ *p* < 0.05, significantly different from control cells; * *p* < 0.05, significantly different from LPS-treated cells.

In order to ascertain whether chestnut extracts are able to exert their protective effect at the transcriptional level, BV-2 cells pretreated with chestnut extracts were exposed to LPS, as previously reported, then mRNA quantification of the inflammatory markers was evaluated by RT-PCR (Figure 7).

As shown in Figure 7A, all chestnut extracts tested at the concentration of 0.5 mg/mL were able to significantly decrease mRNA levels of IL-1β, a potent pro-inflammatory cytokine that is crucial for host-defense responses to infection and injury.

In addition, all the tested extracts were also able to reduce the mRNA level of TNF-α, as reported in Figure 7B. Since it has been reported that TNF-α can induce necrotic or apoptotic cell death [38], it is conceivable that these results agree with the observed protective effect exerted by the chestnut extracts on BV-2 viability.

**Figure 8:**
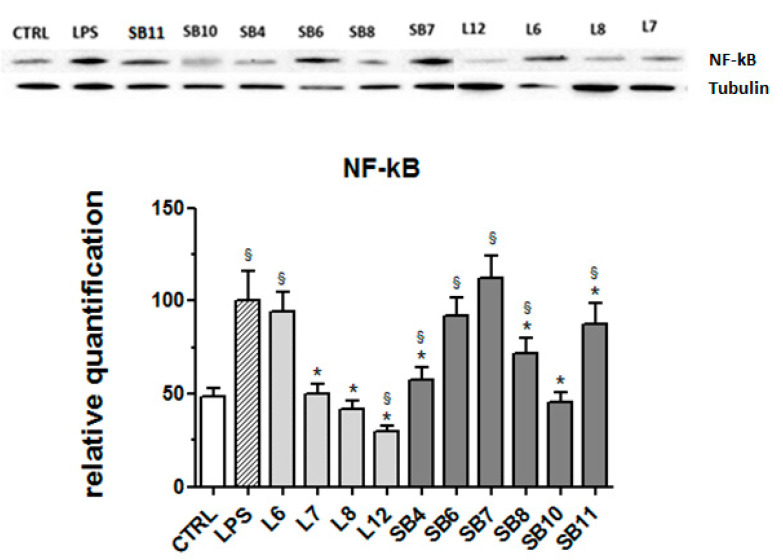
Effect of extracts from chestnut by-products on the NF-kB expression in LPS-treated BV-2 cells. BV-2 cells were incubated for 3 h with 0.5 mg/mL of extracts from chestnut leaves L or spiny burs SB; then, 0.5 μg/mL LPS was added and the cells were incubated for 21 h. Cells were then lysed and the proteins were extracted, separated by SDS-PAGE, transferred to a nitrocellulose membrane, and immunoassayed using anti-NF-kB and anti-tubulin antibodies, as described in the Materials and Methods section. Results are expressed as means ± SD of three independent experiments. Statistical analysis was performed by Bonferroni multiple comparison test following one-way ANOVA. ^§^ *p* < 0.05, significantly different from control cells; * *p* < 0.05, significantly different from LPS-treated cells.

The signaling pathway involving the transcription factor NF-kB is considered a typical pro-inflammatory pathway, largely based on the activation of NF-kB by pro-inflammatory agents and on the role of NF-kB in the expression of pro-inflammatory genes including cytokines, chemokines, and adhesion molecules [39]. For this reason, the expression of this protein in LPS-stimulated BV-2 cells was evaluated in the absence and presence of chestnut extracts. Results obtained by Western blot analysis and reported in Figure 8 reveal that different tested chestnut extracts, with the exceptions of L6 (“Lisanese”), SB6 (“Lisanese”), and SB7 (“Pastanese Biffoni”), are able to significantly decrease the LPS-induced expression of NF-kB.

## 3. Materials and Methods

### 3.1. Chemicals and Materials

Deuterium oxide (H2O-d2, 99.90% D) and MeOH-d4 (99.80% D) were purchased from Eurisotop (Cambridge Isotope Laboratories, Inc, Saint-Aubin, France). Ultra-low Endotoxin FBS was obtained from Euroclone (Euroclone, Milan, Italy). Mini-PROTEAN^®^ TGX™ precast gels 4–20%, Precision Plus Protein™ Unstained Standards, Clarity™ Western ECL Substrate and DC™ protein assay were purchased from Bio-Rad Laboratories (Hercules, California, United States). Primary antibodies against Nf-kB were purchased from Millipore (Merck Millipore, Burlington, Massachusetts, United States). Standard 3-(trimethylsilyl)-propionic-2,2,3,3-d4 acid sodium salt (TMSP), sodium phosphate dibasic anhydrous sodium phosphate monobasic anhydrous, Dulbecco’s modified Eagle medium (DMEM), penicillin, streptomycin, glutamine, lipopolysaccharide (LPS) from *Escherichia coli* serotype O127:B8, 3-(4,5-dimethylthiazol-2-yl)-2,5-diphenyl tetrazolium bromide (MTT), and all the other solvents and chemicals were purchased from Sigma-Aldrich Co. (St. Louis, MO, USA).

### 3.2. Sampling and NMR Metabolomics

Samples were collected during October 2018 in the experimental grove of Granaglione (Bologna, Italy, 44.1589851, 10.90389823). About twenty leaves were collected from each tree, immediately frozen in liquid nitrogen, then stored at −80 °C until they were freeze-dried, ground, and kept in a fridge at 4 °C before the analysis. Spiny burs were dried in a stove at 60 °C, ground, and stored at room temperature. Pomological analysis was performed by Dr. Luca Dondini from the University of Bologna.

Thirty milligrams of freeze-dried and powdered leaves or spiny burs material were extracted using 1 mL of a bland (1:1) MeOH-*d*_4_/H_2_O-*d*_2_ (containing 0.1 M phosphate buffer and 0.01% of TMSP standard). Samples were exposed to ultrasonic waves in a water bath (TransSonic TP 690, Elma, Germany) at a frequency of 35 kHz for 30 min and subsequently centrifuged for 20 min at 1700× *g* (Eppendorf Centrifuge 5804R, Hamburg, Germany); the supernatant (700 µL) was then separated from the pellet and transferred into NMR tubes.

### 3.3. Pre-Purification of Flavonoids from Leaves

The following procedure was designed according to Mandrone et al. [15] with slight modifications.

Seventy-eight grams of powdered chestnut leaves, obtained from all twenty-five lots, were extracted with 1 L of MeOH/H_2_O (1:1). After 30 min of sonication, the extract was centrifuged for 20 min (2469× *g*). Then, it was filtered on a Büchner funnel and dried by a rotary evaporator at 40 °C (R215, Buchi, Flawil, Switzerland).

The extract was suspended in 250 mL of H_2_O and 50 mL of MeOH and subsequently extracted by liquid/liquid partition with 250 mL of hexane, chloroform, ethyl acetate, and *n*-butanol (four times for each solvent).

Dried Et-FR (200 mg) was sub-fractionated by an MPLC instrument (Reveleris^®^, Büchi, Flawil, Switzerland) equipped with a UV-detector and fraction collector and by using a C_18_ column (4 g) at a flow rate of 8 mL/min to collect fractions for UV peaks and setting the UV-Vis detector at 210 nm, 260 nm, and 350 nm. The extract was eluted with a gradient of H_2_O with 0.1% of TFA (solvent A) and MeOH (solvent B). Initial conditions were 5% followed by a linear increase of 5–25% B in 2 min, isocratic elution with 25% B for 5 min, increase to 60% B in 2 min, 60% B for 15 min, increase of 60–70% B in 2 min, 70% B for 15 min, increase of 70–100% B in 2 min, and finally 100% B for 5 min. This procedure resulted in thirteen fractions (from FR1 to FR13). Fraction FR6 contained a mixture of different flavonoids.

### 3.4. NMR and MS Spectra Measurement

^1^H NMR spectra, J-resolved (J-res), ^1^H-^1^H homonuclear, and inverse detected ^1^H-^13^C correlation experiments were recorded at 25 °C on a Varian Inova 600 MHz NMR instrument (600 MHz operating at the ^1^H frequency) equipped with an indirect triple resonance probe. CD_3_OD was used for an internal lock. For ^1^H NMR profiling, the relaxation delay was 2.0 s, observed pulse 5.80 µs, number of scans 256, acquisition time 16 min, and spectral width 9595.78 Hz (corresponding to δ 16.0). For the aqueous samples, a presaturation sequence (PRESAT) was used to suppress the residual H_2_O signal at δ 4.83 (power = −6dB, presaturation delay 2 s).

ESI-MS analyses were performed by direct injection of MeOH solutions of the compounds using a WATERS ZQ 4000 (Milford, MA, USA) mass spectrometer.

### 3.5. NMR Processing and Multivariate Data Treatment

Free induction decays (FIDs) were Fourier transformed, and the resulting spectra were phased, baseline-corrected, and calibrated to TMSP at δ 0.0. Spectral intensities were reduced to integrated regions of equal width (δ 0.04) corresponding to the region from δ 0.0 to 10.0, with scaling on standard at δ 0.0 using the NMR Mestrenova software (Mestrelab Research, Santiago de Compostela, Spain). The analysis of the ^1^H NMR profiles was performed based on an in-house library and comparison with the literature [17,25].

The regions of δ 4.9–4.8 and 3.34–3.26 were excluded from the analysis of the aqueous samples because of the residual solvents’ signals. For multivariate analysis, the model OPLS-DA (Orthogonal Partial Least Squares Discriminant Analysis) was developed using SIMCA-P+ software (v. 15.0, Umetrics, Umeå, Sweden). Data were normalized for standard (at δ 0.0) and subjected to Pareto scaling. The model was evaluated by the goodness of fit (*R*^2^*x*(Cum)) and goodness of prediction (*Q*^2^(Cum)), together with the parameters given by the cross validation tests: permutation test (performed using 30 permutations) and CV-ANOVA [40].

### 3.6. Cell Culture

BV-2 murine microglial cells were kindly provided by Prof. Elisabetta Blasi (University of Modena and Reggio Emilia, Modena, Italy) and were cultured in DMEM supplemented with 10% heat inactivated Ultra-low Endotoxin FBS (Euroclone, Milano, Italy), L-glutamine (1%), and streptomycin (1%) in a humidified incubator maintained at 37 °C and 5% CO_2_, according to Blasi et al. [41].

### 3.7. Cell Viability

Cell viability was evaluated by the MTT assay as previously reported [42]. BV-2 cells were treated with increasing concentrations of extracts from chestnut by-products (0.1–1 mg/mL) for 3 h in 96-well plates and then 0.5 μg/mL LPS was added and the co-treatment prolonged until 24 h. At the end of the treatments, the exhausted medium was eliminated and the MTT solution was added. The blue-violet formazan salt crystals that formed were dissolved with DMSO. The absorbance at 595 nm was measured using a multiwell plate reader (VICTOR^3^ Multilabel Counter; PerkinElmer, Wellesley, MA, USA).

### 3.8. RT-PCR Analysis

The transcriptional level of inflammatory markers was evaluated by RT-PCR as previously reported [42].

After 3 h of treatment with extracts from chestnut by-products (0.5 mg/mL) and 21 h co-treatment with LPS (0.5 μg/mL), total RNA was extracted from BV-2 cells using an RNeasy Mini kit (Qiagen). RNA quantification was performed using a NanoVue spectrophotometer and mRNA was reverse-transcribed into cDNA using iScript cDNA synthesis kit (Bio-Rad). The PCR was carried out in a total volume of 10 μL containing cDNA, SsoAdvanced SYBR Green mix (Bio-Rad), and primers, according to manufacturer’s instructions.

(IL-1β: FW_GTTCCCATTAGACAACTGCACTACAG RV_GTCGTTGCTTGGTTCTCCTTGTA; TNF-α: FW_CCCCAAAGGGATGAGAAGTTC RV_CCTCCACTTGGTGGTTTGCT; GAPDH*: FW_ACCACAGTCCATGCCATCACRV_TCCACCACCCTGTTGCTGTA; *reference control)

### 3.9. Western Blot Analysis

The protein expression of NF-kB was evaluated by Western Blotting as previously reported [43].

After 3 h treatment with extracts from chestnut by-products (0.5 mg/mL) and 21 h co-treatment with LPS (0.5 μg/mL), BV-2 cells (250,000 cells/well) were washed with ice-cold PBS and lysed with RIPA buffer containing a protease and phosphatase inhibitor mixture. Protein concentration of the lysates was determined by Bio-Rad DC protein assay. Proteins (10 μg per lane) were electrophoretically separated on precast gels (Bio-Rad—Laboratories Inc.) and transferred to nitrocellulose membranes. Then, the nitrocellulose membranes were blocked and incubated overnight with primary antibodies (anti-NF-kB or anti-Tubulin I as internal normalizer) at 4 °C. Nitrocellulose membranes were washed with T-TBS and incubated at room temperature for 1 h with secondary antibodies in T-TBS. Chemiluminescence detection was performed using Clarity Western ECL substrate. Bands were acquired with a CCD imager (ChemiDoc MP System, Bio-Rad) and relative densitometric analyses were performed using Image Lab analysis software (Bio-Rad).

## 4. Conclusions

In this study, leaves and spiny burs of *Castanea sativa* from the experimental chestnut grove of Granaglione (Italy) were subjected to phytochemical analysis and tested for potential neuroprotective and anti-inflammatory activities.

The ^1^H NMR-metabolomic analysis performed on ‘Marrone’ and ‘Castagna’ leaves showed that it was possible to distinguish these two classes of samples on the basis of some phytochemical features. In particular, ‘Marrone’ was characterized by lower amounts of all the metabolites, and specifically aromatic compounds, in particular flavonoids, namely, astragalin, isorhamnetin glucoside, and myricitrin, identified by means of NMR and MS experiments. The developed multivariate data model (OPLS-DA), based on the leaves metabolomic profile, might be useful in support of the pomological analysis commonly performed to distinguish ‘Marrone’ and ‘Castagna’, or to discriminate among them when nuts are not available.

With the aim of valorizing the by-products of the experimental chestnut grove, the potential neuroprotective effect of leaves and spiny burs were evaluated in a microglial model. The most current research is focused on the development of neuroprotective therapies aimed at contrasting neuroinflammation at the glial level [44].

On this basis, the effects of extracts on BV-2 cell viability were assayed. Afterwards, their protective activity was assessed in the microglia model exposed to LPS, an inducer of inflammation.

Deepening the study, the effect of extracts on the transcriptional levels of some genes that are protagonists of the inflammatory process, namely IL-1β, TNF-α, and NF-kB, were also evaluated.

Despite the differences found in the metabolomic profiles, leaves and spiny burs of both ‘Marrone’ and ‘Castagna’ at concentrations of 0.1 and 0.5 mg/mL all showed interesting cytoprotective and anti-inflammatory activity on microglia cells, also reducing the expression of the abovementioned genes.

These results represent an important step to encourage the recycling and valorization of *Castanea* by-products, favoring the circular economy and reducing the environmental impact related to management of chestnut grove waste.

Further studies are ongoing to deeply investigate the metabolites that are active in counteracting neuroinflammation.

## Supplementary Materials

The following are available online at https://www.mdpi.com/2218-1989/10/10/408/s1, Figure S1: Structure of the main flavonoids identified in *Castanea* extracts (A) and assigned J-res spectrum of the fraction in which they were contained (B), Figure S2: HMBC spectrum highlighting diagnostic ^13^C-^13^C correlations of myricitrin (A). Superimposed spectra HSQC (green and purple dots) and HMBC (red dots) highlighting other important correlations to detect myricitrin in the analyzed fraction, Figure S3: Superimposed HMBC (red dots) and HSQC (green dots) spectra highlighting diagnostic correlations of isorhametin glucoside, Figure S4: COSY spectrum of the flavonoids containing a fraction in which it is highlighted the characteristic correlation between the protons of the B ring, Table S1: NMR references for flavonoids identified in *Castanea sativa* leaves extract.
