# Supplementary material for: Leaves and Spiny Burs of Castanea Sativa from an Experimental Chestnut Grove: Metabolomic Analysis and Anti-Neuroinflammatory Activity
Source: Metabolites. 2020 Oct 13;10(10):408. doi: 10.3390/metabo10100408 (PMC7601974; doi:10.3390/metabo10100408)
Supplement: Supplementary file 1 [file metabolites-10-00408-s001.pdf]

Article

Leaves and Spiny Burs of *Castanea Sativa* from an Experimental Chestnut Grove: Metabolomic Analysis and Anti-neuroinflammatory Activity

Ilaria Chiocchio [1,†], Cecilia Prata [1,†], Manuela Mandrone [1,*], Fortuna Ricciardiello [2], Pasquale Marrazzo [2], Paola Tomasi [1], Cristina Angeloni [3], Diana Fiorentini [1], Marco Malaguti [2], Ferruccio Poli [1] and Silvana Hrelia [2]

[1] Department of Pharmacy and Biotechnology, Alma Mater Studiorum—University of Bologna, Via Irnerio 48, 40126 Bologna, Italy; ilaria.chiocchio2@unibo.it (I.C.); cecilia.prata@unibo.it (C.P.); paola.tomasi3@unibo.it (P.T.); diana.fiorentini@unibo.it (D.F.); ferruccio.poli@unibo.it (F.P.)

[2] Department for Life Quality Studies, Alma Mater Studiorum—University of Bologna, Corso d'Augusto 237, 47921 Rimini, Italy; fortun.ricciardiello@studio.unibo.it (F.R.); pasquale.marrazzo2@unibo.it (P.M.); marco.malaguti@unibo.it (M.M.); silvana.hrelia@unibo.it (S.H.)

[3] School of Pharmacy, University of Camerino, Via Gentile III da Varano, 62032 Camerino, Italy; cristina.angeloni@unicam.it

* Correspondence: manuela.mandrone2@unibo.it; Tel.: +39-0512091294; Fax +39-051242576

† These authors equally contributed to the manuscript.

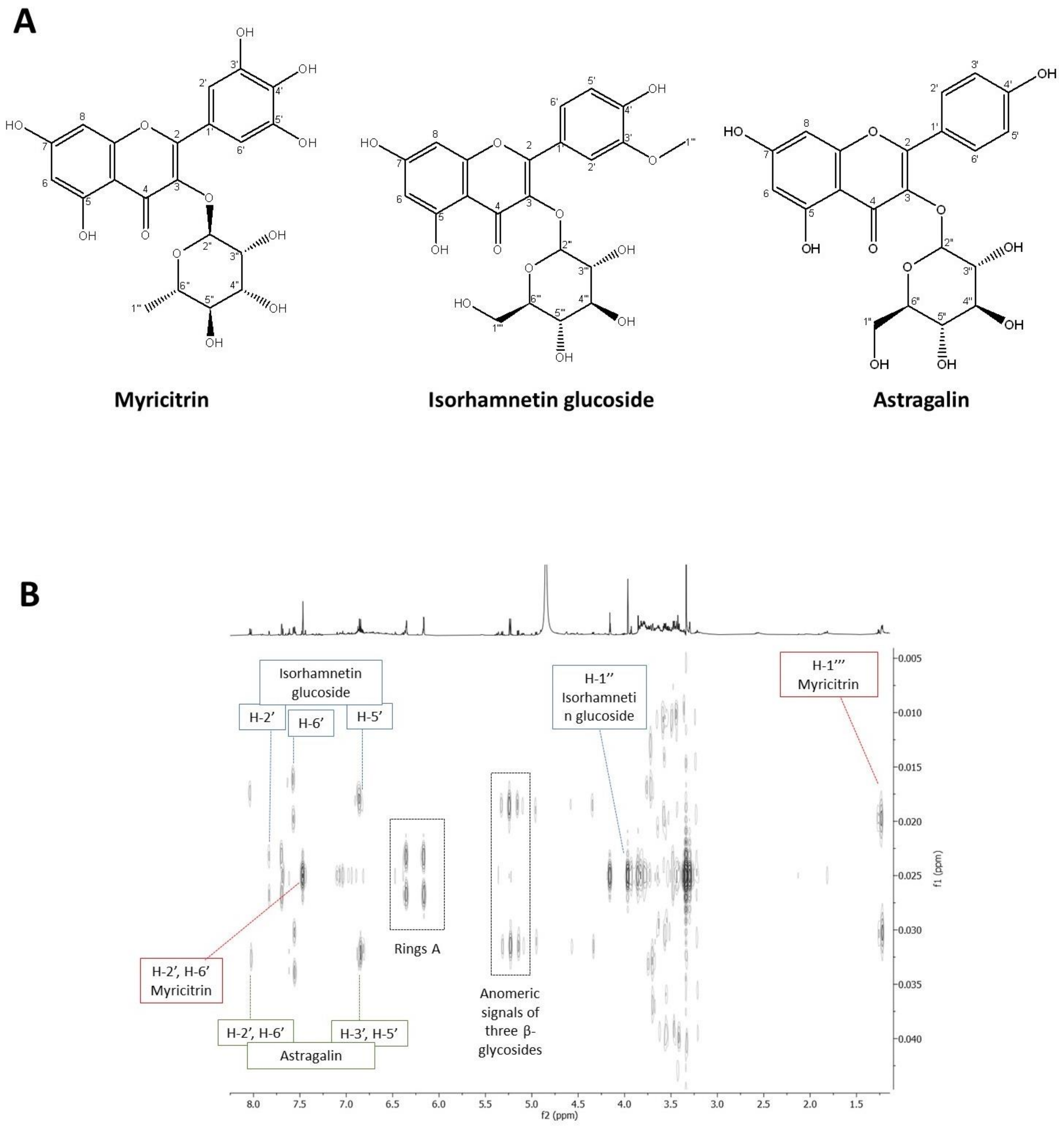

Figure 1. Structure of the main flavonoids identified in *Castanea* extracts (**A**) and assigned J-res spectrum of the fraction in which they were contained (**B**).

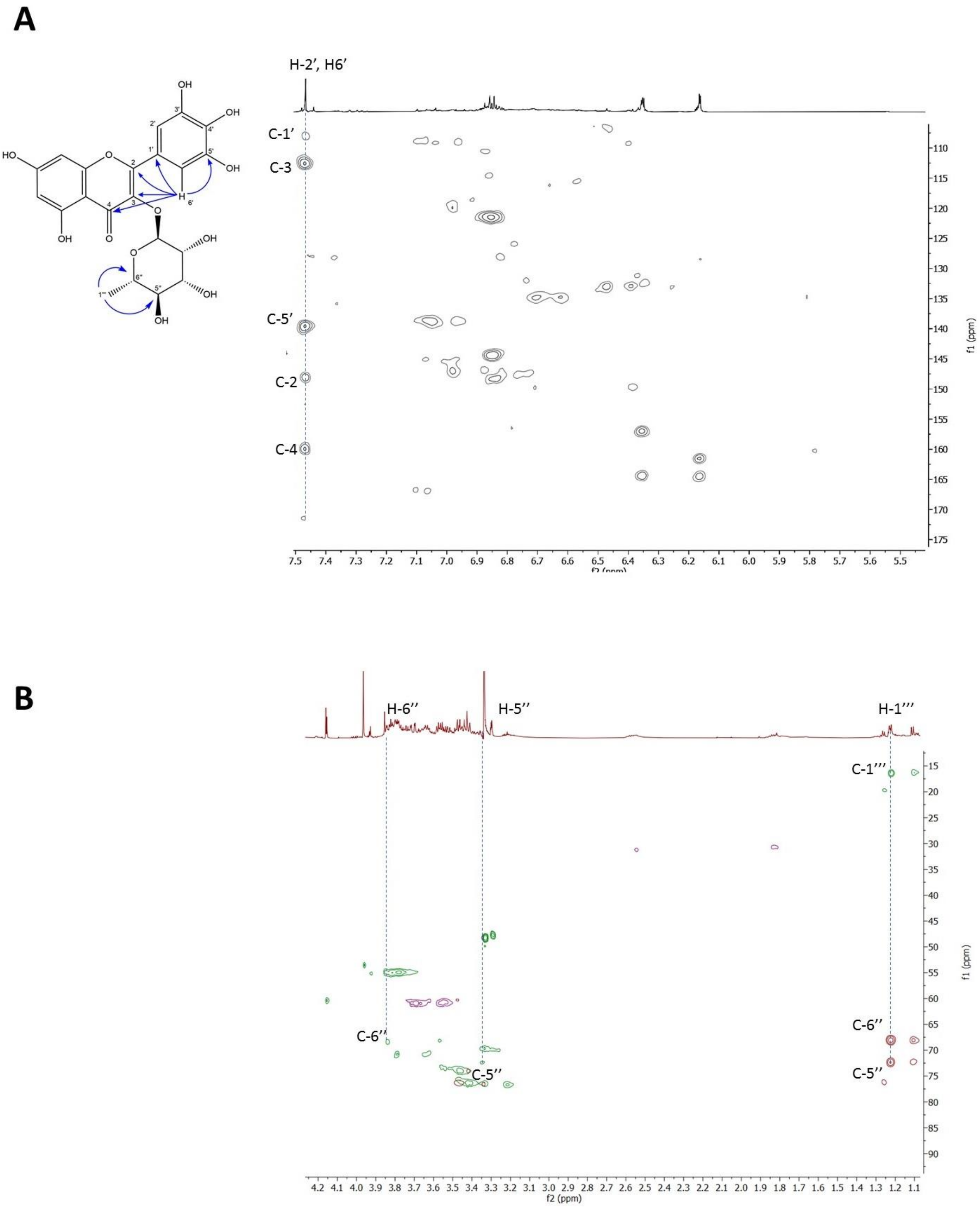

Figure 2. HMBC spectrum highlighting diagnostic ^{13}C-^{13}C correlations of myricitrin (**A**). Superimposed spectra HSQC (green and purple dots) and HMBC (red dots) highlighting other important correlations to detect myricitrin in the analyzed fraction.

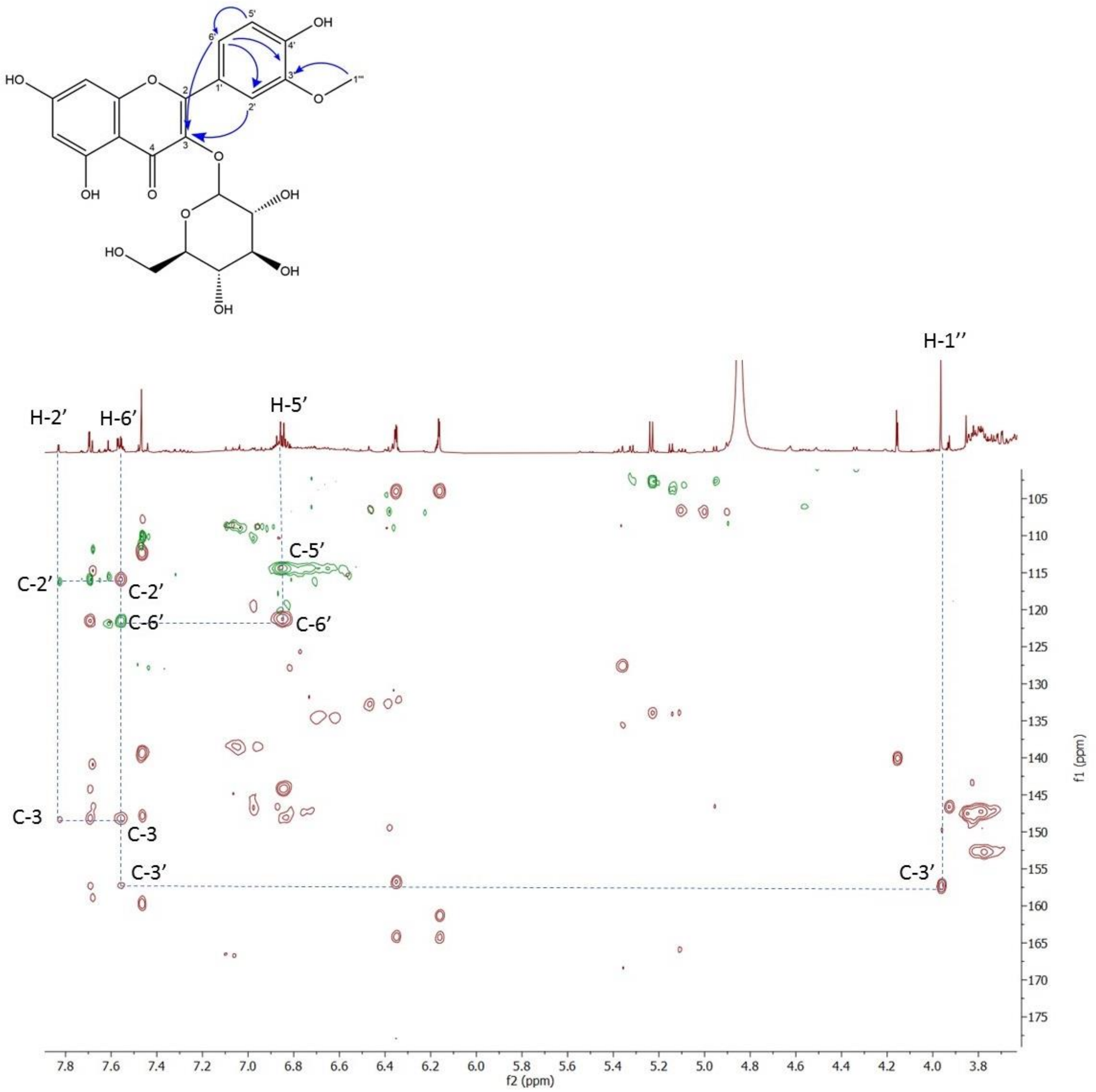

Figure 3. Superimposed HMBC (red dots) and HSQC (green dots) spectra highlighting diagnostic correlations of isorhametin glucoside.

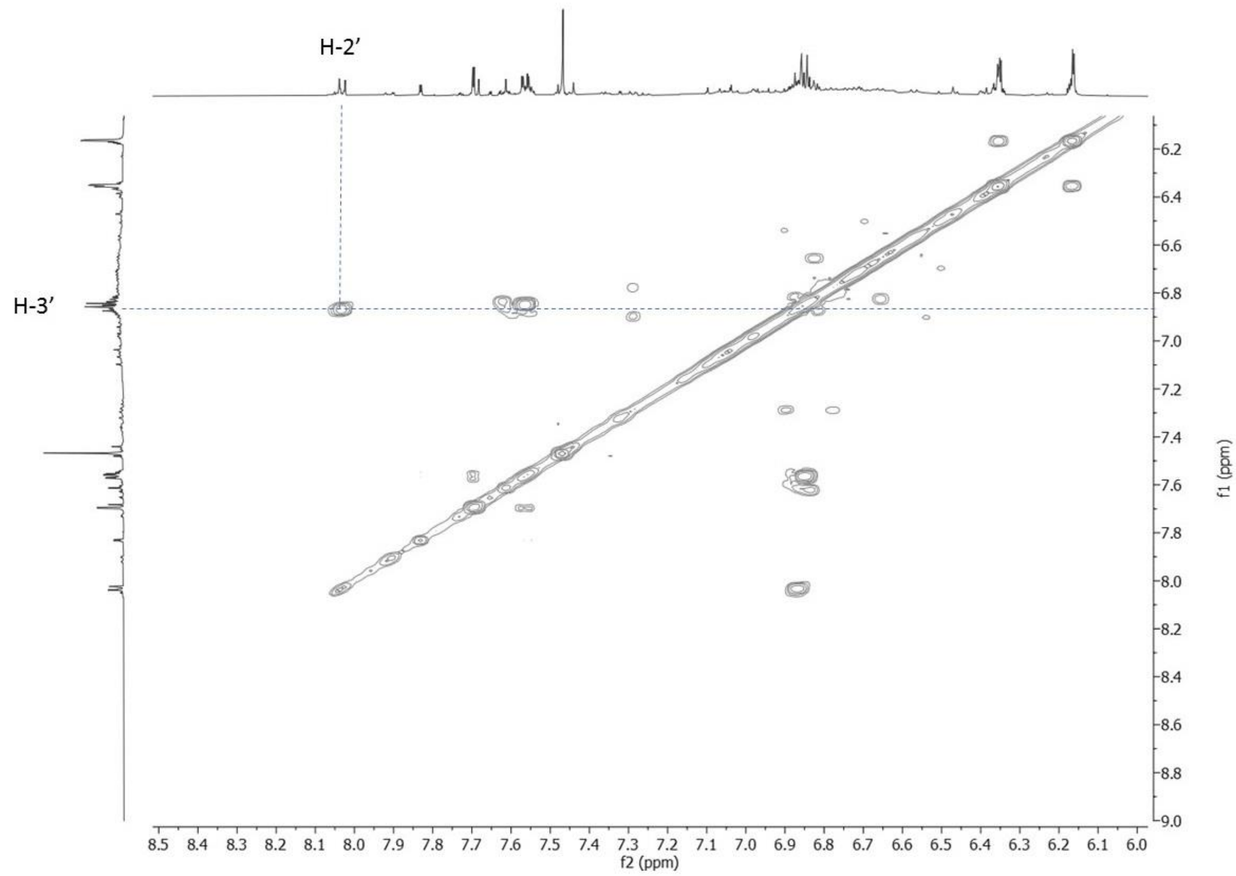

Figure 4. COSY spectrum of the flavonoids containing fraction in which it is highlighted the characteristic correlation between the protons of the B ring.

Compound	Position	^{1}H (δ)	^{13}C (δ)
Astragalin	1′		121.14
	2′, 6′	8.03 (d, J=8.95 Hz)	130.76
	3′, 5′	6.87 (d, J=8.95 Hz)	114.13
	4′		159.95
Isorhamnetin glucoside	2′	7.69 (d, J=2.17 Hz)	115.96
	3′		157.44
	4′		148.12
	5′	6.85 (d, J=8.48 Hz)	114.39
	6′	7.56 (dd, J=2.17, 8.48 Hz)	121.51
	OCH_3	3.91 (s)	53.58
Myricitrin	1′		112.4
	2′, 6′	7.46 (s)	110.29
	3′, 5′		147.73
	4′		139.36

Table 1. NMR references for flavonoids identified in Castanea sativa leaves extract.
